# Stabilization of 1D solitons by fractional derivatives in systems with quintic nonlinearity

**DOI:** 10.1038/s41598-021-04292-7

**Published:** 2022-01-10

**Authors:** V. A. Stephanovich, W. Olchawa

**Affiliations:** Institute of Physics, University of Opole, Oleska 48, 45-052 Opole, Poland

**Keywords:** Nonlinear phenomena, Solitons, Bose-Einstein condensates

## Abstract

We study theoretically the properties of a soliton solution of the fractional Schrödinger equation with quintic nonlinearity. Under “fractional” we understand the Schrödinger equation, where ordinary Laplacian (second spatial derivative in 1D) is substituted by its fractional counterpart with Lévy index $\alpha$. We speculate that the latter substitution corresponds to phenomenological account for disorder in a system. Using analytical (variational and perturbative) and numerical arguments, we have shown that while in the case of Schrödinger equation with the ordinary Laplacian (corresponding to Lévy index $\alpha =2$), the soliton is unstable, even infinitesimal difference $\alpha$ from 2 immediately stabilizes the soliton texture. Our analytical and numerical investigations of $\omega (N)$ dependence ($\omega$ is soliton frequency and *N* its mass) show (within the famous Vakhitov–Kolokolov criterion) the stability of our soliton texture in the fractional $\alpha <2$ case. Direct numerical analysis of the linear stability problem of soliton texture also confirms this point. We show analytically and numerically that fractional Schrödinger equation with quintic nonlinearity admits the existence of (stable) soliton textures at $2/3<\alpha <2$, which is in accord with existing literature data. These results may be relevant to both Bose–Einstein condensates in cold atomic gases and optical solitons in the disordered media.

## Introduction

A self-evident truth is that so-called fractional derivatives describe non-Gaussian probability distributions. The former derivatives, in particular, generate so-called Lévy stable distributions^1–4^. The peculiar feature of these distributions is the power-law asymptotics of their probability densities

1 $$\begin{aligned} f_\alpha (x) \sim |x|^{-1-\alpha } \end{aligned}$$

with $0<\alpha < 2$ being the Lévy index. The power-law decay (1) means that Lévy stable distributions fall off slower than Gaussian ones. The case $\alpha =2$ does not subject to the asymptotics (1) and corresponds to ordinary Gaussian distribution.

When considering the systems with different kinds of nonlinearity that originate from various physical problems (like nonlinear optics, Bose-Einstein condensation (BEC), theory of elasticity^5–7^) the so-called nonlinear Schrödinger equation (NLSE) is usually invoked. To name a few, this equation had been successfully applied to the systems with cubic, quintic as well as mixed (i.e. cubic-quintic) and saturable nonlinearities^6–10^. The NLSE plays an important role in sciences (not only physical), where solitons (in short, nonlinear solitary waves, appearing due to balance of nonlinearity and dispersion, given by the spatial derivatives) appear^8,11^. In nonlinear optics, the NLSE is a basic tool to investigate light propagation in nonlinear optical media^5–7^. Furthermore, the famous Gross–Pitaevskii equation (GPE), which is widely used to describe the mean field properties of BEC in ultracold bosonic gases^12^, is indeed the NLSE with cubic nonlinearity. The above permits to use NLSE to theoretically predict and study many unusual and experimentally relevant nonlinear phenomena (like soliton textures) in optical, condensed-matter and atomic physics setups^6,7^.

The one-dimensional (1D) NLSE with quintic nonlinearity stands alone as it describes the Tonks–Girardeau regime, which occurs for Bose gas with repulsive interaction in one dimension only and has many unusual physical implications like “fermionization” of bosons^13,14^. The experimental realization of the latter condition has been done in Ref.^14^ employing a gas of ultracold rubidium atoms in a two-dimensional optical lattice. In the opposite case of attractive interaction, the quintic 1D NLSE has exact soliton solution^9^. This solution, however, is unstable. Namely, according to Vakhitov–Kolokolov (VK) criterion^15^, it has only marginal stability, i.e. it is neither stable nor unstable, see Refs.^9,10^. Moreover, this marginal stability occurs for only one value of the soliton frequency, for its infinitesimal changes it either collapses or decays^9,10^. The most common method to stabilize such soliton texture is to use so-called optical lattice (a spatially periodic polarization pattern, formed by counter-propagating laser beams) or external parabolic potential^9,10^. The first time consideration of the stable solitons propagation in the optical lattice for the case of fractional NLSE, has been reported in the papers^16,17^. In these papers, the ordinary Laplacian (second spatial derivative in 1D case) in NLSE had been substituted by the fractional one. Those solitons were formed as a result of joint action of the Kerr-type (third degree) nonlinearity and fractional dispersion.

Here we show that the same replacement of ordinary Laplacian by fractional one stabilizes the soliton texture even without optical lattice. This is achieved even at infinitesimal “fractionality” corresponding to the deviation of Lévy index from “ordinary case” $\alpha =2$.

The above replacement of ordinary Laplacian by the fractional one is being widely used in so-called fractional quantum mechanics, introduced by Laskin^18–20^. The possibility of such replacement had been derived by Feynman path integral application to random elementary trajectories, obeying Lévy (instead of ordinary Brownian trajectories with Gaussian statistics) statistics^18^. In other words, in fractional quantum mechanics, the ordinary Feynman path integral over Brownian trajectories is replaced by that over so-called Lévy flights. The stochastic trajectories of Lévy flights can be thought of as Brownian ones, interspersed by some continuous jumps, which can sometimes be extremely long. These jumps, actually, lead to the discontinuities (say, disordering) in the initial Brownian paths so that the resulting probability densities obey the above Lévy stable distributions with power-law tails (1). As non-Gaussian distributions usually describe the situations with the disorder (like the strong crystal lattice distortions leading to the translational symmetry loss), here we can also speak about the disorder. In this case, as Lévy index $\alpha$ is responsible for the deviation of the underlying system trajectories from Gaussian ones, this quantity plays a role of a phenomenological descriptor of the degree of disorder. The Schrödinger equation with fractional Laplacian had been called fractional Schrödinger equation^18–20^.

In the linear regime, the 1D problems of fractional quantum well^21^ and quantum oscillator^22^ had been solved. Possible physical realizations of 2D^23^ and 3D hydrogen atom^24^ as well as 2D hydrogen atom with Rytova–Keldysh (screened) interaction^25^ had been pointed out along with solutions of corresponding problems. It has been speculated there (see Ref.^25^) that as strong (like substance amorphization) disorder is usually associated with non-Gaussian probability distributions, the fractional Schrödinger equation gives the simple phenomenological description of corresponding strongly disordered quantum system.

When nonlinear terms are introduced into the fractional Schrödinger equation, we are dealing with fractional NLSE. Such equations (both in 1D and higher dimensions) had been widely investigated theoretically both in 1D and higher dimensions, see^26,27^ and references therein. The purpose of the present paper is to analyze analytically (using perturbation approach near $\alpha =2$ and variationally) and numerically the stabilization of the soliton texture in the simplest possible (without cubic terms, external potential, etc) model^9^ by the introduction of the fractional derivatives in the corresponding NLSE. As an introduction of the fractional derivatives in NLSE may be related to the disorder, we may call our finding *the soliton stabilization by disorder*.

Note, that although fractional Laplacian is defined for Lévy indices $0<\alpha <2$ (see also above), in fractional quantum mechanics^18–20^, the solutions of the corresponding Schrödinger equation in one spatial dimension exist for $1<\alpha <2$ due to the localization property of wave functions. The latter property is related to the fact that in quantum mechanics the wave function should be normalizable, i.e. the integral square of its modulus should be equal to one. This imposes restrictions on the wave function decay at infinities and implies the range of existence of the solutions at $1<\alpha <2$. This is not the case for nonlinear solitonic problems, where the existence of soliton solutions with respect to $\alpha$ is dictated by the balance of nonlinearity and dispersion (now fractional) only. A latter balance had been studied in detail in the mathematical work^28^, where the existence of soliton solutions for fractional NLSE with arbitrary power-law nonlinearity had been proved. Our variational results show that stable soliton textures exist in fractional NLSE with quintic nonlinearity at $2/3<\alpha <2$, which is in agreement with mathematical literature^28^. We discuss also the possible implications of soliton texture stabilization by the disorder, which is described phenomenologically by the fractional derivatives.

The rest of the paper is organized as follows. In the next section “The model”, we introduce the model and its qualitative analysis, including stability questions. In the two following sections “Perturbation theory” and “Variational treatment. Lévy index limits for soliton solution existence” sections we study our model perturbatively (around “ordinary” case $\alpha =2$) and variationally. The latter treatment gives the limits of existence of a soliton texture in terms of the Lévy index $\alpha$. “Numerical calculations” section is completely devoted to the numerical studies of our soliton textures. This includes comprehensive direct numerical studies of a linear soliton stability. We end in “Outlook” section with a discussion of the realistic physical systems, where our results are applicable as well as their possible generalizations.

## The model

In the spirit of what was said above, here we consider the substitution of the ordinary Laplacian (second derivative in one spatial dimension) in the quintic NLSE of the form

2 $$\begin{aligned} i\frac{\partial \psi }{\partial t}=-\frac{\partial ^2 \psi }{\partial x^2}+\chi |\psi |^4\psi . \end{aligned}$$

Here, $\psi =\psi (x,t)$ is a wave function and parameter $\chi$ distinguishes between repulsive (focusing) $\chi >0$ and attractive (defocusing) $\chi <0$ nonlinearities. Our aim is to substitute the ordinary second spatial derivative in (2) by the 1D fractional Laplacian

3 $$\begin{aligned} |\Delta |^{\alpha /2}g(x)= & {} A_\alpha \int _{-\infty }^{\infty }\frac{g(u)-g(x)}{|u-x|^{1+\alpha }}du, \end{aligned}$$

4 $$\begin{aligned} A_\alpha= & {} \frac{\Gamma (1+\alpha )}{\pi }\sin \frac{\pi \alpha }{2}, \end{aligned}$$

where $0<\alpha <2$ is the Lévy index. The fractional Laplacian (3) is Riesz fractional derivative, see Ref.^29^ for details. We note that at $\alpha =2$ the fractional 1D Laplacian converts into ordinary second spatial derivative. The details of the corresponding transition can be found, for instance in Ref.^30^. The easiest way to check that is via Fourier transformation, which for second derivative gives $-k^2$. This implies that the Fourier image of the fractional Laplacian (3) gives $-|k|^\alpha$ or explicitly

5 $$\begin{aligned} |\Delta |^{\alpha /2}f(x)=-\frac{1}{2\pi }\int _{-\infty }^\infty |k|^\alpha f(k)e^{-ikx}dk. \end{aligned}$$

The expression (5) will be used in subsequent calculations.

The equation, having soliton solution for $\chi <0$, is obtained from (2), (3) by setting

6 $$\begin{aligned} \psi (x,t)=y(x)e^{i\omega t}, \end{aligned}$$

where $\omega$ is a soliton frequency. This generates following fractional equation for *y*(*x*)

7 $$\begin{aligned} A_\alpha \int _{-\infty }^{\infty }\frac{y(p+x)-y(x)}{|p|^{1+\alpha }}dp-\omega y +|\chi | y^5=0, \end{aligned}$$

where $A_\alpha$ is defined by (4). To derive the equation (7), we made substitution $p=u-x$ in the integral (3). At $\alpha =2$ the equation (7) transforms into

8 $$\begin{aligned} y_0''-\omega y_0+|\chi |y_0^5=0, \end{aligned}$$

which has the exact soliton solution^9^

9 $$\begin{aligned} y_0(x)=\frac{A}{\sqrt{\cosh Bx}},\ A=\left( \frac{3\omega }{|\chi |}\right) ^{1/4},\ B=2\sqrt{\omega }. \end{aligned}$$

If we calculate the norm of this soliton solution

10 $$\begin{aligned} N_0=\int _{-\infty }^\infty y_0^2dx=\frac{\pi }{2}\sqrt{\frac{3}{|\chi |}}, \end{aligned}$$

we see that it is independent of soliton frequency $\omega$ so that VK stability criterion (necessary condition for soliton texture stability^15^) $dN/d\omega <0$ does not hold in this case. Rather, here we have $dN/d\omega =0$, i.e. the texture is marginally stable.

Below we shall show numerically that the VK criterion $dN/d\omega <0$ corresponds to the exponential decay of the linear corrections to the soliton texture for $\alpha <2$. In other words, in our 1D fractional case, the above analytical form of the VK criterion correctly reflects the soliton stability.

Moreover, as it had been shown in Ref.^10^, this marginal stability holds only for a single value of the norm $N=N_0$ (10). At $N>N_0$ the soliton (9) collapses, while for $N<N_0$ it decays into the ground state $y=0$^10^. This behavior can be well understood qualitatively by the simple calculation of powers in corresponding energy functional. Namely, the energy functional, corresponding to the Eq. (8), has the form

11 $$\begin{aligned} W_0=\int _{-\infty }^\infty \left[ -\frac{1}{2} (y')^2-\omega \frac{y^2}{2}+\frac{|\chi |}{6}y^6\right] dx. \end{aligned}$$

If we consider for a while the solution $y_0(x)$ as a trial function (with inverse localization radius *B* being variational parameter and fixed norm $N=\pi A^2/B$) and substitute it to the functional (11). This yields

12 $$\begin{aligned} E_0(B)=-\frac{N}{16}B^2-\frac{1}{2} \omega N+\frac{|\chi |N^3}{12\pi ^2}B^2. \end{aligned}$$

In the expression (12), first term comes from the “kinetic energy” (dispersion term), i.e. first term in the integrand (11). It is seen that the energy $E_0$ (12) can be represented in the form $E_0=\varkappa B^2-\omega N/2$, where $\varkappa =\frac{|\chi |N^3}{12\pi ^2}-\frac{N}{16}$. This immediately shows that the energy $E_0$ has extremum at $B=0$ (corresponding to infinite localization radius, i.e. actually to the already decayed state of the soliton), but maximum or minimum depends on the sign of the coefficient $\varkappa$. Namely, at $\varkappa >0$ we have minimum (at $B=0$) and at $\varkappa <0$ - maximum. The condition $\varkappa =0$ occurs just at $N=N_0$ (10), while $\varkappa \gtrless 0$ corresponds to $N \gtrless N_0$, giving rise to above behavior, i.e. collapse at $N>N_0$ and decay at $N<N_0$. At the same time, the texture (9) corresponds to the case of $\varkappa =0$, i.e. “no parabola”, which means neither maximum nor minimum or aforementioned marginal stability. Note that this reasoning is already very rough as extremum at $B=0$ already corresponds to the decayed state with infinite localization radius.

It is instructive to compare the above results with the case of cubic nonlinearity, corresponding to the substitution of the nonlinear term $|\chi |y_0^5$ in the equation (8) by $gy_0^3$. The contribution from this term into $E_0$ is $BN^2/(2\pi )$ so that the energy has extremum at finite $B_{extr}=4gN/\pi$, i.e. at finite value of the soliton localization radius, proportional to its norm. This shows that the case of quintic nonlinearity has kind of degeneracy as the contributions coming from dispersion and nonlineariry have the same power. At the same time, for cubic nonlinearity this degeneracy is absent. On the other hand, the soliton in this case is also unstable as $E_0$ has a maximum.

This means, that it is harder to stabilize the soliton in the system with pure quintic nonlinearity as here we should first “gain” the finite soliton radius, where its energy has an extremum. Below we show that the fractional derivatives can well cope with this task.

## Perturbation theory

We begin with perturbational treatment of possible soliton solution of the fractional equation (7) as it can be considered to be “exact” in the range of validity of corresponding perturbation theory. In other words, perturbational treatment does not imply any trial function, but has the exact solution (8) as its zeroth approach.

The latter fact implies that the small parameter $\varepsilon$ of our perturbation treatment is the “fractionality”, i.e. deviation of the Lévy index $\alpha$ from 2:

13 $$\begin{aligned} \varepsilon =2-\alpha . \end{aligned}$$

To expand the solution of the equation (7) over small parameter $\varepsilon$ (13), we represent it in the form

14a $$\begin{aligned} \left[ \frac{d^2}{dx^2}+\widehat{\Delta F}\right] y(x)-\omega y(x) +|\chi | y(x)^5=0, \end{aligned}$$

where

14b $$\begin{aligned} \widehat{\Delta F}y(x)= & {} A_\mu \int _{-\infty }^{\infty }\frac{y(p+x,t)-y(x,t)}{|p|^{1+\alpha }}dp-y''\equiv \nonumber \\\equiv & {} \frac{1}{2\pi }\int _{-\infty }^\infty \left( k^2-|k|^\alpha \right) y(k)e^{-ikx}dk. \end{aligned}$$

We have further

14c $$\begin{aligned} k^2-|k|^\alpha= & {} k^2-|k|^{2-\varepsilon }=k^2\left( 1-e^{-\varepsilon \ln |k|}\right) \approx \nonumber \\\approx & {} \varepsilon k^2\ln |k|-\frac{\varepsilon ^2}{2!}k^2\ln ^2|k|+\cdots , \end{aligned}$$

We see that at $\alpha \rightarrow 2$, the fractional derivative generates logarithmic corrections to the initial “dispersion law” $\sim k^2$. Note, that as at $k \rightarrow 0$ $k^2\ln ^n k \rightarrow 0$, the corresponding integrals at $k=0$ are all convergent. The Eqs. (14a)–(14c) can now be recast to the form, suitable for perturbative solution. We have

15 $$\begin{aligned} y''-\omega y+|\chi |y^5= & {} -\widehat{\Delta F}y \equiv -\frac{1}{2\pi }\int _{-\infty }^\infty y(k) \times \nonumber \\&\times \bigg [\varepsilon k^2\ln |k|-\frac{\varepsilon ^2}{2!}k^2\ln ^2|k|+\cdots \bigg ]e^{-ikx}dk. \end{aligned}$$

We look for the perturbative solution of (15) in the form

16 $$\begin{aligned} y(x)=y_0(x)+\varepsilon y_1(x)+\varepsilon ^2 y_2(x)+\cdots , \end{aligned}$$

where $y_0(x)$ is defined by (8). In this case, the right hand side of Eq. (15) assumes the form

17 $$\begin{aligned} -\widehat{\Delta F}y\approx & {} \alpha _1\varepsilon +\alpha _2\varepsilon ^2+\alpha _3\varepsilon ^3+ \cdots \end{aligned}$$

18 $$\begin{aligned} \alpha _1= & {} -\frac{1}{2\pi }\int _{-\infty }^\infty y_0(k) k^2\ln |k|e^{-ikx}dk,\nonumber \\ \alpha _2= & {} -\frac{1}{2\pi }\int _{-\infty }^\infty \bigg [y_1(k)\ln |k|-\nonumber \\&-\frac{1}{2} y_0(k)\ln ^2|k|\bigg ]k^2e^{-ikx}dk. \end{aligned}$$

It is seen that, as usually in the perturbation theories, the expressions for higher-order corrections become progressively more cumbersome. Now, in zeroth approach we have, as expected, $\alpha =2$ ($\varepsilon =0$) so that $y=y_0$ is defined by the expression (9). In the first approach $y=y_0+\varepsilon y_1$ we have

19 $$\begin{aligned} y_1''-y_1\bigg (\omega -5|\chi |y_0^4\bigg )=-\frac{1}{2\pi }\int _{-\infty }^\infty y_0(k) k^2\ln |k|e^{-ikx}dk, \end{aligned}$$

while the second approach $y=y_0+\varepsilon y_1+\varepsilon ^2y_2$ yields

20 $$\begin{aligned} & y_{{2^{{{\prime \prime }}} }} - y_{2} (\omega - 5\left| \chi \right|y_{0}^{4} ) = - 10\left| \chi \right|y_{1}^{2} y_{0}^{3} - \frac{1}{{2\pi }} \\ & \quad \times \int_{{ - \infty }}^{\infty } {\left[ {y_{1} (k)\ln \left| k \right| - \frac{1}{2}y_{0} (k)\ln ^{2} \left| k \right|} \right]} k^{2} e^{{ - ikx}} dk. \\ \end{aligned}$$

It turns out that the equations for each perturbative correction $y_n$ to the solution $y_0$ (9) can be solved in quadratures. Namely, it can be shown (this is also seen from the equations (19), (20) for two first corrections), that the general structure of the equation for each perturbation correction has the form

21 $$\begin{aligned} \hat{L}y_n(x)=g_n(x),\ \hat{L}= & {} \frac{d^2}{dx^2}-\omega \bigg (1-\frac{5|\chi |}{\omega }y_0^4\bigg )\equiv \nonumber \\\equiv & {} \frac{d^2}{dx^2}-\omega \bigg (1-\frac{15}{\cosh ^2Bx}\bigg ), \end{aligned}$$

where we have already substituted $y_0$ (9). The properties of operator $\hat{L}$ in (21) are already known, see, e.g.^31^. This means that we can look for solution of the equations for perturbative corrections (21) in the form of expansion over the eigenfunctions of operator $\hat{L}$. But as this operator has also continuous spectrum, latter approach becomes much less profitable than simple method of variations of parameters, known from classical theory of linear differential equations, see, e.g.^32^. To realize the latter method, we need first to find two linearly independent solutions of the homogeneous equation $\hat{L} y=0$. It is well known that one of the fundamental solutions of the homogeneous equation (21) is always the derivative of the unperturbed soliton solution $y_0$ (9). It has following explicit form

22 $$\begin{aligned} u_1(x)=\frac{\sinh Bx}{\cosh ^{3/2}Bx}. \end{aligned}$$

As it is also well-known from the theory of linear differential equations, the second linearly independent solution $u_2(x)$ can be found as

23 $$\begin{aligned} u_2(x)=u_1(x)\int \frac{dx}{u_1^2(x)}=\frac{\cosh ^2Bx -2}{\cosh ^{3/2}Bx}. \end{aligned}$$

It is seen from expressions (22), (23) that $u_1(x)$ is odd and decaying function, while $u_2(x)$ is even and growing function. General solution of the inhomogeneous equation (21) assumes the form

24 $$\begin{aligned} y_n(x)= & {} C_{1n}(x)u_1(x)+C_{2n}(x)u_2(x), \end{aligned}$$

25 $$\begin{aligned} C_{1n}(x)= & {} -\frac{1}{B}\int _{-\infty }^x u_2(x)g_n(x)dx,\nonumber \\ C_{2n}(x)= & {} \frac{1}{B}\int _{-\infty }^x u_1(x)g_n(x)dx. \end{aligned}$$

Our numerical calculations show that the functions $C_{1n}(x)u_1(x)$ are odd, while $C_{2n}(x)u_2(x)$ are even. As our soliton texture should be even function, we take the even term only, i.e.

26 $$\begin{aligned} y_n(x)=C_{2n}(x)u_2(x). \end{aligned}$$

**Figure 1:**
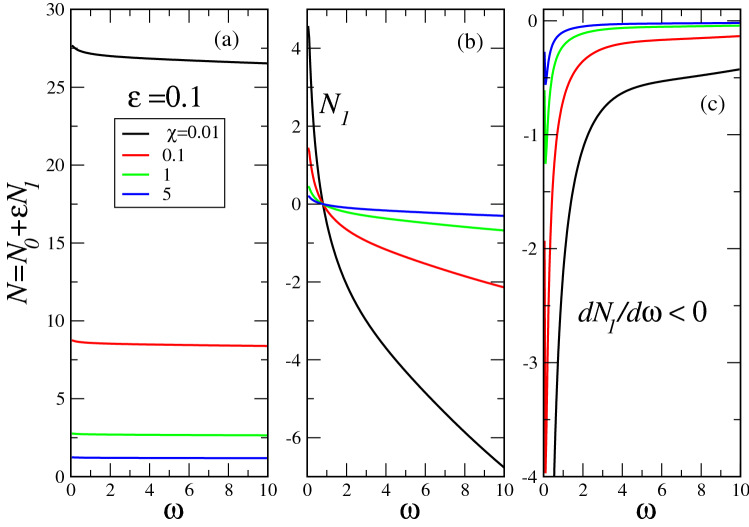
The “fractional soliton” norm, calculated in the first order of perturbation theory at several nonlinearity parameters $\chi$ and $\alpha =1.9$ ($\varepsilon =0.1$, legend in panel (**a**)). As first order corrections (panel (**b**)) are negative at $\omega >1$, panel (a) shows the overall norm, which turns out to be positive as it should be. Panel (**c**) shows the derivatives $dN/d\omega \equiv dN_1/d\omega$, which are negative. At large $\omega$ the corrections at any $\chi$ sease to depend on $\omega$ so that $dN_1/d\omega \rightarrow 0$ at $\omega \rightarrow \infty$. The larger $|\chi |$, the more pronounced is latter behavior.

The obtained perturbative solution of our problem permits to calculate the soliton characteristics near $\alpha =2$. As soliton norm

27 $$\begin{aligned} N=\int _{-\infty }^\infty |\psi (x,t)|^2dx\equiv \int _{-\infty }^\infty y(x)^2dx \end{aligned}$$

plays a major role in the VK soliton stability criterion, we calculate it to obtain

28 $$\begin{aligned} N= & {} N_0+\varepsilon N_1+\varepsilon ^2 N_2+\cdots ,\nonumber \\ N_1= & {} 2\int _{-\infty }^\infty y_0y_1dx,\nonumber \\ N_2= & {} \int _{-\infty }^\infty \left( y_1^2+2y_0y_2\right) dx, \end{aligned}$$

where $N_0$ is defined by (10). In the spirit of VK criterion, as $N_0$ does not depend on $\omega$, the stability will be determined by the signs of $dN_1/d\omega$ and higher order corrections. As $dN_1/d\omega$ is the largest term (it is proportional to $\varepsilon$, while subsequent terms are proportional to powers of latter small parameter), the stability would primarily be determined by $dN_1/d\omega$ sign. Our numerical calculations (based on inhomogeneous solution (26)) in the first order of perturbation theory show that this is indeed the case, i.e. $dN_1/d\omega <0$. This situation is portrayed in Fig. 1. It is clearly seen that small “fractionality” (i.e. small deviation of $\alpha$ from 2, corresponding in this specific case to $\varepsilon =0.1$) indeed stabilizes the soliton, making its norm to be $\omega$ dependent. The panel (a) of the Figure shows that total norm is positive, while first order correction (even at relatively large $\varepsilon =0.1$) is smaller then $N_0$. Figure 1 shows a remarkable property of fractional derivatives: even small “fractionality” stabilizes the initially unstable soliton. This point will be further confirmed below by direct numerical simulation of the soliton texture stability.

## Variational treatment. Lévy index limits for soliton solution existence

In the case of “ordinary” soliton with $\alpha =2$, the variational functional has the well-known form (11), where the square of 1D gradient (first spatial derivative) being varied, gives the second spatial derivative in the resulting differential equation (8). Our analysis shows that the fractional analog of the first term in the integrand (11) has the form of the square of the fractional gradient

29 $$\begin{aligned} -\frac{1}{2} \left( |\nabla |^{\alpha /2}y(x)\right) ^2. \end{aligned}$$

The most convenient way to define the fractional gradient is through its Fourier transform, namely

30 $$\begin{aligned} |\nabla |^{\alpha /2}y(x)=-\frac{1}{2\pi }\int _{-\infty }^\infty |k|^{\alpha /2} y(k)e^{-ikx}dk. \end{aligned}$$

With respect to the above, the variational functional for fractional case can be written in the form

31 $$\begin{aligned} W_\alpha= & {} -\frac{1}{2} \int _{-\infty }^\infty \left( |\nabla |^{\alpha /2}y(x)\right) ^2 dx-\nonumber \\&-\frac{\omega }{2} \int _{-\infty }^\infty y^2(x)dx+ \frac{|\chi |}{6}\int _{-\infty }^\infty y^6(x)dx. \end{aligned}$$

It turns out that many general properties of soliton texture (like minimal Lévy index $\alpha _{min}$, at which the soliton solution of the Eq. (7) seaces to exist) can be obtained without knowledge of explicit functional form of the trial function (although this will be done subsequently for the sake of quantitaive comparison with perturbative and numerical results), but rather of more general “scaling” form of it. Namely, we consider trial function of the form

32 $$\begin{aligned} y(x)=Af(Bx), \end{aligned}$$

where *A* and *B* are variational parameters. The contribution of the first term in (31) can be calculated most conveniently via the Fourier image *y*(*k*) of the function *y*(*x*). We have

33 $$\begin{aligned} y(k)= & {} A\int _{-\infty }^\infty f(Bx)e^{ikx}dx=\nonumber \\= & {} \frac{A}{B}\int _{-\infty }^\infty f(t)e^{i\frac{k}{B}t}dt\equiv \frac{A}{B}\tilde{y}\left( \frac{k}{B}\right) . \end{aligned}$$

Substitution of the inverse Fourier image (30) of the function (33) into the integrand of the first term in (31) generates factor $e^{-ix(k+k')}$ in it. After integration over *x* this yields $2\pi \delta (k+k')$, where $\delta (z)$ is Dirac $\delta$ function. After integration over $k'$ we than arrive at the following expression for the first term

34 $$\begin{aligned} I_{1\alpha }= & {} -\frac{1}{2\pi }\frac{A^2}{B^2}\int _0^\infty k^\alpha {\tilde{y}}^2\left( \frac{k}{B}\right) dk \equiv \nonumber \\\equiv & {} -\lambda _\alpha A^2B^{\alpha -1}, \ \lambda _\alpha =\frac{1}{2\pi }\int _0^\infty z^\alpha {\tilde{y}}^2(z)dz. \end{aligned}$$

Here we used the fact that Fourier image (33) is the even function of its argument. The final expression for variational energy then reads

35 $$\begin{aligned} E_\alpha =-\lambda _\alpha A^2B^{\alpha -1}+\frac{\langle f^6 \rangle }{6B}\left[ |\chi |A^6-3\omega A^2\frac{\langle f^2 \rangle }{\langle f^6 \rangle }\right] , \end{aligned}$$

where $\langle f^n \rangle =\int _{-\infty }^\infty f^n(x)dx$. In this case soliton norm

36 $$\begin{aligned} N=\frac{A^2}{B} \langle f^2 \rangle . \end{aligned}$$

As the functions $\langle f^2 \rangle$ and $\langle f^6 \rangle$ are all positive and independent of *A* and *B*, we can find the extremum of $E_\alpha$ (35) with respect to these parameters. After lengthy calculations we arrive at following result

37 $$\begin{aligned} A_0^4=\frac{\langle f^2 \rangle }{2\langle f^6 \rangle }\frac{6\alpha \omega }{|\chi |(3\alpha -2)},\ B_0^\alpha =\frac{\omega \langle f^2 \rangle }{\lambda _\alpha (3\alpha -2)}. \end{aligned}$$

As $A_0^4$ and $B_0^\alpha$ should be positive, the function $E_\alpha$ has extremum at $3\alpha -2>0$ or $\alpha >2/3$. Thus we arrive at the following criterion of soliton solution existence

38 $$\begin{aligned} \frac{2}{3}<\alpha <2. \end{aligned}$$

This criterion coincides with that obtained from rigorous mathematical treatment of the localized solutions of the fractional differential equations with $2p+1$ degree nonlinearity in *d* dimensional space^28^. In the paper^28^ this criterion relates the degree of nonlinearity *p* with space dimensionality *d* and critical (lower) Lévy index $\alpha _{cr}$. In our notations this criterion reads $\alpha _{cr}=pd/(1+p)$, which for $p=2$ and $d=1$ gives $\alpha _{cr}=2/3$. Below we shall demonstrate the correctness of criterion (38) by direct numerical solution of Eq. (7).

Note that the expression (36) with respect to (37) permits to obtain the explicit expression for $N(\omega )$ in fractional case. Substitution of (37) into (36) yields

39 $$\begin{aligned} N= & {} N_0\omega ^{\frac{1}{2}-\frac{1}{\alpha }},\nonumber \\ N_0= & {} (3\alpha -2)^{\frac{1}{\alpha }-\frac{1}{2}}\lambda _\alpha ^{\frac{1}{\alpha }} \langle f^2 \rangle ^{\frac{3}{2}-\frac{1}{\alpha }} \left( \frac{3\alpha }{|\chi | \langle f^6 \rangle } \right) ^{\frac{1}{2}}. \end{aligned}$$

The expression (39) shows immediately, that as soon as $\alpha$ becomes less then 2 (fractional case), *N* acquires the dependence on $\omega$, thus giving nonzero derivative $dN/d\omega$. As $N_0>0$, the function $N(\omega )$ (39) is decreasing so that $dN/d\omega =N_0(\frac{1}{2}-\frac{1}{\alpha }) \omega ^{-\frac{1}{2}-\frac{1}{\alpha }}<0$ at $\alpha <2$. This shows that our variational (actually scaling as no explicit form of trial function has been used) approach predicts the fractional soliton stability according to VK criterion. This is one more chief result of the present consideration, which will be confirmed below by direct numerical simulations.

The above shows that simple scaling arguments, put in the context of a variational method, permit to capture correctly the soliton “phase diagram”, i.e. the range of Lévy indices, where the localized solution exists. Moreover, our analysis shows that the extremum (37) corresponds to the minimum, which permits to hope that our soliton texture is stable within the range (38) of admissible $\alpha$ values. Our explicit numerical solution of the fractional equation (7) along with simulations of the soliton texture stability will show that this is indeed the case.

To further study the character of extremum in the fractional case $\alpha <2$, we, similar to the case $\alpha =2$ investigate the dependence $E_\alpha (B)$ at fixed *N*. For that we eliminate *A* from the expression (35) with the help of the relation (36) to get

40 $$\begin{aligned} E_\alpha (B)=-\frac{\lambda _\alpha N}{\langle f^2 \rangle }B^\alpha +\frac{|\chi |}{6}\frac{\langle f^6 \rangle }{\langle f^2 \rangle ^3}N^3B^2-\frac{\omega N}{2}. \end{aligned}$$

The structure of the expression (40) reveals the fact that as the contributions coming from dispersion ($\sim B^\alpha$) and nonlinearity ($\sim B^2$) have different exponents, this immediately implies that contrary to the case $\alpha =2$ (12), we have extremum at finite soliton radius 1/*B*. To be specific, the condition $dE_\alpha (B)/dB=0$ generates following extremizing value

41 $$\begin{aligned} B_{min}^{\alpha -2}=\frac{|\chi |N^2}{3\alpha \lambda _\alpha }\frac{\langle f^6 \rangle }{\langle f^2 \rangle ^2}. \end{aligned}$$

Lower index “min” in the expression (41) means that the extremum corresponds to minimum at $\alpha <2$. This can be easily checked by substitution of the extremizing value (41) into the second derivative of $E_\alpha (B)$ (40). We have $E''(B_{min})=u(2-\alpha )$, where $u=|\chi |N^3\langle f^6 \rangle /(3\langle f^2 \rangle ^3) >0$. This shows that at fixed norm the variational energy (40) has a minimum at finite value of soliton radius (41) for all admissible values (38) of Lévy index $\alpha$.

## Numerical calculations

To check how both perturbational and variational methods work, it is instructive to compare them with the direct numerical solution of the Eq. (7). For that, we choose the expression (9) as a trial function with *A* and *B* being variational parameters. This gives $\langle f^2 \rangle =\pi /2$, $\langle f^6 \rangle =\pi /4$ and $\lambda _\alpha$ (34) to be calculated numerically for each $\alpha$. The substitution

42 $$\begin{aligned} y(x)=|\chi |^{-1/4}z(x) \end{aligned}$$

permits to obtain for *z*(*x*) the equation, which does not contain $\chi$. In other words, the equation for *z*(*x*) is similar to Eq. (7) for $|\chi |=1$. This means that in our numerical calculations, we can well use the latter equation. The solutions for other $\chi$’s can be easily obtained from those for *z*(*x*) with the help of the substitution (42).

**Figure 2:**
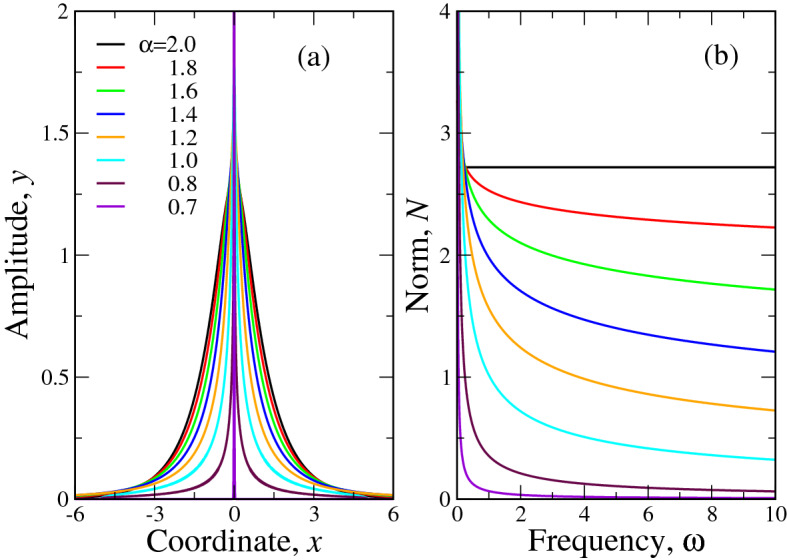
Panel (**a**). The numerical solution of the fractional equation (7) for $\omega =1$ and $|\chi |=1$, corresponding to the function *z*(*x*) (42). Different values of Lévy index $\alpha$ are coded by colors and shown in the legend of the panel (**a**). Panel (**b**). The “fractional soliton” norm, calculated for the same $\alpha$ and $0<\omega <10$. It is seen that while in “ordinary” ($\alpha =2$) case *N* does not depend on $\omega$, as soon as $\alpha <2$, the decreasing dependence $N(\omega )$ is acquired so that $dN/d\omega <0$ and soliton stabilizes according to VK criterion.

The solutions of the equation (7) for $|\chi |=1$ and $\omega =1$ are portrayed in Fig. 2a for different $\alpha$. The shape of solutions at other $\omega$ is qualitatively similar to those at $\omega =1$. It is seen that as $\alpha$ approaches the critical value $\alpha _{cr}=2/3 \approx 0.667$, the solution becomes progressively more peaked and at $\alpha \rightarrow \alpha _{cr}$, the peak height goes to infinity. In other words, at $\alpha = \alpha _{cr}=2/3$ we have “fractionality collapse” of the soliton. We may speculate here, that as Lévy index $\alpha$ can be viewed as a measure of disorder in a system (see^3,23–25^ and references therein; the more $\alpha$ deviates from 2, the stronger is the disorder), the value $\alpha _{cr}=2/3$ may be regarded as some threshold disorder strength, at which the solitons cease to exist in a system. Really, at $\alpha < \alpha _{cr}$, our iterative process of the numerical solution diverges so that no localized solutions of the equation (7) could be found.

Latter outcome is in accord with the results of variational treatment (38). This shows that our variational treatment captures well the “phase diagram” (range of admissible Lévy index values) of the fractional soliton. Below we will see that it gives also very good (indistinguishable in the scale of plot) approximation to the numerical solution of the fractional equation (7). This shows that this method could be considered as an efficient analytical tool to obtain and study the solution of the soliton equations with fractional derivatives. Note, that as no explicit expression for trial function has been introduced (see (32)), the above variational method is not sensitive to the trial function details and thus could be of general nature. This means that it can be well applied for more complex models of “fractional” solitons, like those with mixed or saturated nonlinearities.

The numerical dependence $N(\omega )$ is reported in Fig. 2b. The coincidence with expression (39) is clearly seen: while at $\alpha =2$ the norm *N* is independent of $\omega$, this dependence appears at $\alpha <2$. Moreover, as this dependence is decreasing function, $dN/d\omega <0$, which shows numerically, that according to VK criterion, the soliton becomes stable in fractional case $\alpha <2$.

**Figure 3:**
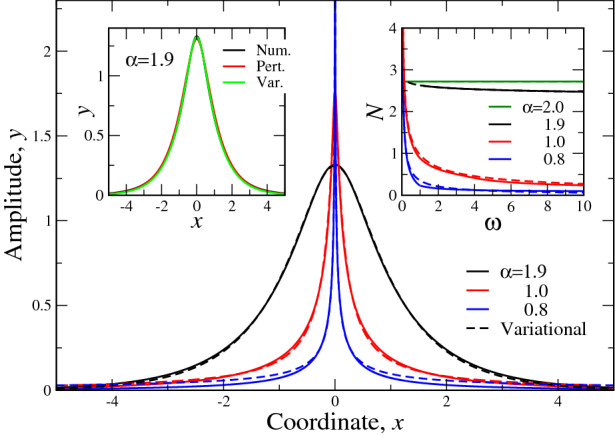
Main panel. Comparison of numerical (full lines) and variational (dashed lines) solutions of the fractional soliton equation (7) for different Lévy indices $\alpha$, coded by colors and shown in the legend. Here, without loss of generality, we put $\omega =1$ and $|\chi |=1$, see Eq. (42). Left inset. Comparison of numerical (black), perturbational (red) and variational (green) solutions of (7) for $\alpha =1.9$ ($\varepsilon =0.1$, Eq. (13)). All three curves are almost indistinguishable in the scale of the plot. Right inset reports the comparison of numerical and variational (39) (with the trial function in the form of (9)) dependencies $N(\omega )$. Full lines—numerical curves, dashed lines—variational. The values of Lévy indices $\alpha$ are shown in the legend.

We now compare the variational, perturbational (in the range of validity, i.e. at $\alpha$ close to 2) and numerical results for both soliton structure and its norm. Such comparison is reported in Fig. 3. The main panel shows the comparison of variational and numerical soliton structures *y*(*x*) at some selected values of $\alpha$. It is seen that the closer is $\alpha$ to 2, the better is a coincidence. On the other hand, for $\alpha$ close to its critical value 2/3, the overall coincidence worsens (although it remains within limits of 5%) especially at the “tails” of the *y*(*x*) function. This is because our simple trial function (7) has exponential asymptotics at infinities, while it is well-known (see, e.g.,^2–4,28,33^) that the asymptotics of the fractional differential equations solutions is usually power-law (1). Latter power-law asymptotics starts to manifest itself for $\alpha$’s somewhere close to 2/3. This means that a more sophisticated, multi-parametric, trial function would improve coincidence with numerics near threshold $\alpha$ value.

Right inset in Fig. 3 reports a comparison of numerical and variational dependences $N(\omega )$, obtained from the corresponding solutions at different $\alpha$. This shows that the variational solution gives a pretty good approximation to the numerical one also at a wide range of the frequencies $0<\omega <10$. The coincidence of $N(\omega )$ curves even at $\alpha$ close to 2/3 (e.g. for $\alpha =0.8$) shows that for integral characteristics, like $N(\omega )$, the details of solution behaviour is not that important. Thus, for soliton characteristics, involving integrals of *y*(*x*), even the simplest possible trial function (9) gives very good coincidence with the numerical curve, which requires a substantial amount of time and computer resources (to calculate $N(\omega )$, we should first solve (7) numerically for each $\omega$) to be obtained.

**Figure 4:**
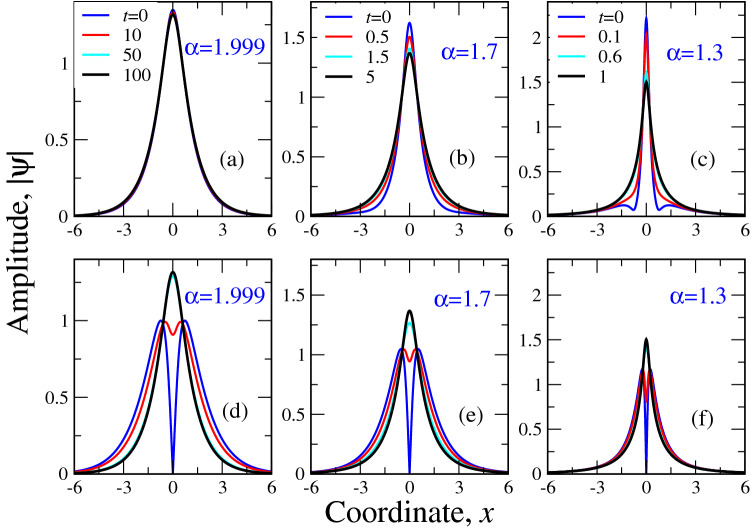
The numerical simulation of the soliton time evolution of the fractional NLSE (2), (3) for $\omega =1$ and $|\chi |=1$ in the form $|\psi (x,t)|\equiv \sqrt{\psi (x,t)\psi ^*(x,t)}$. Here, $\psi (x,t)$ is defined by the expression (45) and comprises the complex perturbation of the static textures (42). Reported are the dependences of $|\psi (x)|$ at different time instants *t*, shown in the panels for different Lévy indices $\alpha$. Upper panels (**a**), (**b**) and (**c**) correspond to the positive (the same sign as unperturbed static solutions (42)) perturbation, while lower ones ((**d**), (**e**), (**f**))—to the negative. Panels (**a**) and (**d**), (**b**) and (**e**) as well as (**c**) and (**f**) correspond to the same Lévy index $\alpha$. Their evolution to the static textures occurs at the same time instants, shown in the corresponding upper panels. At large *t* all solitons evolve to the static textures (42), shown as bold black lines in the panels.

Left inset in Fig. 3 compares numerical, variational and perturbational (up to the first order in small parameter $\varepsilon$ (13)) solutions *y*(*x*). The solutions, obtained at $\alpha =0.9$, are coded by colors and it is seen that they are indistinguishable in the scale of the plot. Our analysis shows that maximal error here is around 0.1%. We choose $\alpha =0.9$ ($\varepsilon =0.1$) for the first order of perturbation theory to be valid. It is obvious, that if we consider also the higher orders in the small parameter $\varepsilon$, we can descent further in $\alpha$. As consideration of the higher orders of perturbation theory is usually associated with the growth of complexity of calculations, the variational solution in this sense is much more profitable. This is because it gives the approximate (as we saw above, the accuracy of the approximation is generally very good) analytical solution of the problem for all admissible values of Lévy index $\alpha$. On the other hand, the perturbational solution can be regarded as “exact” (it does not use any variational *ansätse*) in the range of its validity. This means that perturbational soliton may be considered as a complementary to variational one near $\alpha =2$. This is especially true in view that in the present problem the solution for $\alpha =2$ (zeroth approximation in perturbation theory) (9) is exact.

We are now in a position to study the soliton stability numerically. This is because the direct use of VK result $dN/d\omega <0$ can sometimes adulterate the real (i.e. numerical) stability criterion. This is especially true for the fractional case as the theorem of Vakhitov and Kolokolov^15^ had been stated and proved for the solitons having ordinary second spatial derivative as a dispersion term. To accomplish the above task, we consider the linear stability problem for the soliton solution of the initial fractional NLSE (2) with fractional Laplacian (3). Namely, we consider following substitution

43 $$\begin{aligned} \psi (x,t)=\left[ y(x)+p(x)e^{\lambda t}+q^*(x)e^{\lambda ^* t}\right] e^{i\omega t},\ p,q<<y. \end{aligned}$$

Here *y*(*x*) is undisturbed (by the small perturbations *p* and *q* ) soliton texture (i.e. the above numerical solution of the Eq.  (7)) and asterisk means complex conjugation. Substitution of (43) into fractional NLSE (2), (3) with its subsequent linearization over *p* and *q* generates following eigenvalue problem

44 $$\begin{aligned} \hat{L} p-f(x)q= & {} i\lambda p,\ \hat{L} q-f(x)p=-i\lambda q, \nonumber \\ \hat{L}= & {} -|\Delta |^{\alpha /2}+\omega -3z^4(x),\ f(x)=2z^4(x), \end{aligned}$$

where $-|\Delta |^{\alpha /2}$ is defined by (3) and function *z*(*x*) by (42). By solving numerically the spectral problem (44), it can be shown that our texture *y*(*x*) is stable if all real parts $\lambda _r$ of the eigenvalues $\lambda$ are zero. It turns out that this is the case for the entire domain of $\alpha$ (38) and $\omega$ values. Our analysis shows that the spectrum of imaginary part, $\lambda _i$, consists of negative values only, i.e. $i\lambda _i=-p_n$, where *n* enumerates the eigenvalues. This means that the corrections $\sim e^{\lambda t}=e^{-p_nt}$ (43) to the soliton solution *y*(*x*) decay exponentially so that our soliton texture is stable. At the same time for $\alpha =2$, the imaginary part $\lambda _i$ equals zero so that soliton is marginally stable. Similar to our preceding results, the soliton gains stability at $\alpha <2$ only. Also, the modulus of the smallest (i.e. “most negative”) eigenvalue grows as $\alpha \rightarrow 2/3$. For instance, for $\omega =1$ our numerical calculations show that $p_{min}(\alpha =1.9)=-0.618$ ($p_{min}$ is the smallest eigenvalue so that the correction decays primarily like $e^{-0.618 t}$), $p_{min}(\alpha =1.7)=-1.294$, $p_{min}(\alpha =1.3)=-3.435$, $p_{min}(\alpha =1.0)=-9.162$ and $p_{min}(\alpha =0.74)=-210.1$ the lowest $\alpha$, accessible in our numerical calculations. Most probably, at $\alpha = 2/3$ $p_{min} \rightarrow -\infty$. This means that in fractional case our soliton texture may be prone to collapse at $\alpha = 2/3$ since the collapsed configuration can be considered as “absolutely stable”. However, as in real physical setups one cannot change $\alpha$ (say, it is related to the degree of disorder in a sample), for each specific Lévy index value, the soliton texture is stable.

To demonstrate the considered soliton stability, it is instructive to check it by the direct numerical simulations of the soliton texture time evolution. In the spirit of linear stability *ansats* (43), for our numerical simulations, we consider the following substitution

45 $$\begin{aligned} \psi (x,t)=y(x)+\delta \psi (x,t), \end{aligned}$$

where *y*(*x*) is a static texture (real function), obtained by the numerical solution (42) of the equation (7) for each $\alpha$. In this case, the soliton stability would mean the decay of $\delta \psi (x,t)$ as time goes to infinity. In other words, as time passes, for a stable case, the initial texture tends to *y*(*x*). As function $\delta \psi (x,t)$ is complex, for our pictorial demonstration of the soliton stability, we simulate the dynamics of the modulus $|\psi (x,t)|=\sqrt{\psi (x,t)\psi ^*(x,t)}$. Note, that if we choose too large amplitudes of the real and imaginary parts of the initial perturbation $\delta \psi (x,0)$, the soliton will lose its stability. Namely, it will either collapse or “smear down” into the ground state $\psi =0$. Also, in this case, the numerical method instability may come into play. The criterion of our choice of perturbation $\delta \psi (x,0)$ was that for the “regular” case $\alpha =2$ the texture stays in its initial state for a sufficiently long time $t>500$. Also, our observations show, that the choice of $\delta \psi (x,0)$ in the form, which yields the negative contribution into $\psi (x,t)$, may “stabilize” the soliton so that slightly larger amplitudes of $\delta \psi (x,0)$ are admissible. The results are reported in Fig. 4 for three representative values of Lévy index $\alpha$. In Fig. 4, three upper panels correspond to positive initial perturbations and three lower - to the negative one. To demonstrate, how relaxation to the static case “works”, we intentionally take $\alpha =1.999$ rather than $\alpha =2$. It is seen, that for positive perturbation, the relaxation to the static texture occurs from above, while for negative one from below. The main observation, however, is that the relaxation to the static texture *y*(*x*) occurs quicker at smaller $\alpha$ in accordance with the above stability analysis. Namely, while at $\alpha =1.999$, the relaxation to (both for positive and negative perturbations) *y*(*x*) “finishes” at $t=100$, for $\alpha =1.3$ this time is 100 times smaller, i.e. for $t>1$, we already have the static texture. As we approach the critical value $\alpha =2/3$, this time tends to zero.

## Outlook

The chief message of the present consideration is that the “fractionality” (deviation of Lévy index $\alpha$ from 2), stabilizes the soliton texture. The physical origins of the “fractionality” may be different. Although the explicit discussions of the underlying physical mechanisms are scarce, we can distinguish two main implementations. One is possible solid-state realization, called Lévy crystal^34^. This model deals essentially with the discretization of the fractional Schrödinger equation to obtain the effective tight-binding lattice model, where the hoppings between lattice cites obey Lévy statistics. To generate latter statistics experimentally, hoppings in the lattice must be carefully adjusted, which may be a prohibitively complex task. Another implementation is related to the light dynamics in an optical cavity^35^. Although this is readily realizable physically, the reason for the usage of fractional derivatives in corresponding linear Schrödinger equation was phenomenological, i.e. the specific dispersion law, generated by the fractional derivative, was needed to realize the physical setup. We mention also one more solid-state model, dealing with polariton condensates^36^. In this study, however, the question of the physical origin of “fractionality” had not been considered at all. On the other hand, recently we proposed^23–25^ the possible physical origin (to some extent justification of usage) of “fractionality” coming from the strong (i.e. non-Gaussian) disorder in a system. The main point here^25^ is that in a disordered system we usually have a broad, non-Gaussian distribution of its physical quantities. In quantum mechanics, such strong disorder usually leads to localization of initially (before the introduction of a disorder by, say, doping by some impurities) itinerant states. This is the essence of the famous Anderson localization phenomenon^37^. In the context of our solitonic problem, this means “more localization” of the soliton (for instance its collapse at $\alpha =2/3$) so that it becomes “more stable” than in the ordinary non-fractional case, corresponding to $\alpha =2$. We may call our effect *the soliton stabilization by disorder*.

Although here we consider the simple model with quintic nonlinearity in one spatial dimension, our preliminary consideration shows that this is the case for more complicated models, containing more intricate nonlinearities (like competing cubic-quintic, higher order like septic and/or saturable nonlinearities) as well as external space and time dependent potentials. A noteworthy result is the variational analysis of the problem, based on the scaling argument only. In other words, we take the trial function in the scaling form (32) without any suppositions on its exact shape. The only supposition was that the integrals $\langle f^2 \rangle$ and $\langle f^6 \rangle$ should be convergent. Such scaling analysis permits us not only to establish (known from the mathematical literature, see^28^ and references therein) the range of $\alpha$’s, where the soliton solution exist but to show that already at an infinitesimal deviation of $\alpha$ from 2, the soliton acquires stability according to VK criterion, see Eq. (39). As the conclusion of soliton stability has been confirmed by the numerical analysis, we can assert, that our simple “scaling-variational” procedure is suitable to study also the properties of more complicated soliton textures.

One more important result is the construction of perturbation theory near the ordinary case $\alpha =2$. The point is that such perturbational expansion is the only analytical tool to investigate the soliton textures in models of any complexity when variational ansats is hard to conceive for some reasons. Although perturbational calculations (especially in higher orders) may be very cumbersome, they may serve as guidance for numerical simulations as the nonlocal character of fractional derivatives makes even the numerical calculations tricky. Also, the stability effects (for instance using VK criterion) can be investigated analytically within the latter approach.

An interesting generalization of our fractional soliton problem is a substitution of time derivative in Eq. (2) by its fractional counterpart. Our preliminary analysis shows that such substitution has implications on soliton dynamics and stability. We postpone the discussion of the latter interesting dynamical effects for future publications. The results reported here are relevant for disordered multicomponent and nonlinear optical systems both in 1D and higher dimensions. They also may be used to control (e.g. by varying Lévy index $\alpha$) the properties of Bose-Einstein condensates.

## Methods

The details of our theoretical methodology and those of working with fractional derivatives and fractional Laplacians, in particular, have been described in the sections “The model”, as well as in the two following sections “Perturbation theory” and “Variational treatment. Lévy index limits for soliton solution existence”. The numerical simulations of soliton structure and stability have been conducted using the commercial *Mathematica* software package as well as C++ routines, partially written *ad hoc* and partially taken from the standard libraries like LAPACK.
